# Chronic Compression of the Dorsal Root Ganglion Enhances Mechanically Evoked Pain Behavior and the Activity of Cutaneous Nociceptors in Mice

**DOI:** 10.1371/journal.pone.0137512

**Published:** 2015-09-10

**Authors:** Tao Wang, Olivia Hurwitz, Steven G. Shimada, Lintao Qu, Kai Fu, Pu Zhang, Chao Ma, Robert H. LaMotte

**Affiliations:** 1 Institute of Basic Medical Sciences, Chinese Academy of Medical Sciences, School of Basic Medicine, Peking Union Medical College, Neuroscience Center, Department of Anatomy, Histology and Embryology, Beijing, China; 2 Department of Anesthesiology, Yale University School of Medicine, New Haven, Connecticut, United States of America; 3 Department of Neurosurgery, Neurosurgery Pain Research Institute, Johns Hopkins University, Baltimore, Maryland; Boston Children’s Hospital and Harvard Medical School, UNITED STATES

## Abstract

Radicular pain in humans is usually caused by intraforaminal stenosis and other diseases affecting the spinal nerve, root, or dorsal root ganglion (DRG). Previous studies discovered that a chronic compression of the DRG (CCD) induced mechanical allodynia in rats and mice, with enhanced excitability of DRG neurons. We investigated whether CCD altered the pain-like behavior and also the responses of cutaneous nociceptors with unmyelinated axons (C-fibers) to a normally aversive punctate mechanical stimulus delivered to the hairy skin of the hind limb of the mouse. The incidence of a foot shaking evoked by indentation of the dorsum of foot with an aversive von Frey filament (tip diameter 200 μm, bending force 20 mN) was significantly higher in the foot ipsilateral to the CCD surgery as compared to the contralateral side on post-operative days 2 to 8. Mechanically-evoked action potentials were electrophysiologically recorded from the L3 DRG, in vivo, from cell bodies visually identified as expressing a transgenically labeled fluorescent marker (neurons expressing either the receptor MrgprA3 or MrgprD). After CCD, 26.7% of MrgprA3^+^ and 32.1% MrgprD^+^ neurons exhibited spontaneous activity (SA), while none of the unoperated control neurons had SA. MrgprA3^+^ and MrgprD^+^ neurons in the compressed DRG exhibited, in comparison with neurons from unoperated control mice, an increased response to the punctate mechanical stimuli for each force applied (6, 20, 40, and 80 mN). We conclude that CCD produced both a behavioral hyperalgesia and an enhanced response of cutaneous C-nociceptors to aversive punctate mechanical stimuli.

## Introduction

The compression of a dorsal root ganglion (DRG) and adjacent spinal nerve and root can cause sensory loss but also abnormal sensations, including paresthesias, pain [1, 2] and itch [3]. Animal models of radicular and low back pain include those that compress and/or inflame the tissues near the DRG, spinal root or nerve spinal ganglion [4–7]. In one model, a chronic compression of the DRG (CCD) is produced by the unilateral implantation of a rod into each of two adjacent lumbar intervertebral foramina. The behavioral effects of CCD include an ipsilateral tactile allodynia to innocuous punctate mechanical stimuli (von Frey filaments). Tactile allodynia after CCD is defined by an increased incidence of paw withdrawal to innocuous von Frey filaments that do not elicit this aversive behavior under normal conditions (prior to CCD) [8–11]. Presumably the innocuous stimuli do not elicit sufficient activity in cutaneous nociceptors to elicit pain behavior. However, should these nociceptors become sensitized after CCD one would hypothesize that normally aversive von Frey stimuli would elicit a greater incidence of pain behavior (defined as hyperalgesia). The present study tested the hypothesis that CCD causes mechanical hyperalgesia.

CCD causes an increase in the excitability of the cell bodies of DRG neurons in the rat [8–11] and in the mouse [12, 13]. In the mouse, the CCD-induced spontaneous activity and/or lowered rheobase of small diameter DRG neurons was accompanied, and possibly caused by, an increased expression of tetrodotoxin(TTX)-resistant and TTX-sensitive Na^+^ current [13]. Because these electrophysiological recordings were obtained from an intact, but isolated, DRG in vitro, it was not possible to determine the peripheral receptive field properties of these C-type neurons, for example those that innervate the skin. To date there have been no studies of the effects of CCD on the excitability of the peripheral terminals of nociceptors with unmyelinated axons (C-nociceptors).

The behavioral hypersensitivity to punctate mechanical stimuli (allodynia or hyperalgesia) might be caused by the sensitization of nociceptive neurons in the spinal dorsal horn as a result of CCD induced ongoing (“spontaneous”) action potential (AP) activity in one or more types of cutaneous C-nociceptors. Alternatively, or in addition, enhanced nociceptive behavior to mechanical stimuli after CCD might occur if the mechanical stimuli evoked more APs in the cutaneous C-nociceptors.

A goal of the present study was to test the hypothesis that CCD induces spontaneous APs in C-nociceptors in hairy skin or increases their responses to a normally aversive punctate mechanical stimulus. Our strategy was to record APs extracellularly, in vivo, from visualized single somata in the DRG [14]. A green fluorescent protein (GFP) marker was used to aid in the identification of mechanosensitive C nociceptors. In one type of transgenic mouse the GFP was present in neurons expressing the MrgprD receptor for beta alanine [15] and, in a different type of mouse, in neurons expressing the MrgprA3 receptor for chloroquine [16]. Both types of neurons were previously shown to innervate the stratum granulosum of the epidermis of the hairy skin in the hind paw [16]. Together the two types of Mrgpr-expressing neurons constitute the majority of mechanosensitive C-nociceptors innervating the hairy skin of the hind paw [16–18].

It is likely that the identified population of cutaneous C-mechanonociceptors contribute to mechanically-evoked pain-like behavior evoked by a normally aversive punctate stimulus. Yet mechanosensitive C-nociceptors have also been implicated as "pruriceptors" that mediate chemically-evoked itch in mice [16, 19, 20], monkeys [21] and humans [22]. An aim of the present study was to compare discharges obtained in response to aversive mechanical stimuli with discharges evoked in previous studies by chemical pruritogens. The purpose is to gain insight into a possible mechanism for the differential coding of itch and pain within the responses of the same population of cutaneous nociceptors.

## Materials and Methods

### Animals

For behavioral tests, 11 male, wild type, C57BL6 mice (Charles River, Wilmington, MA) each of 25–30 g-weight were used. In the electrophysiological experiments, two types of transgenic male mice, were used each of 28 to g-weight, having a C57BL6 background, and expressing a green fluorescent protein (GFP) in the cell body of a particular type of cutaneous, mechanosensitive C-nociceptive DRG neurons: a) neurons expressing the MrgprA3 receptor for chloroquine (in "Mrgpr A3^+^ mice") [16], and b) neurons expressing the MrgprD receptor for beta alanine (in "MrgprD^+^ mice") [13]. Electrophysiological recordings were obtained from 9 MrgprA3^+^ mice, 4 of which were unoperated controls and 5 with CCD, and from 11 MrgprD-GFP^+^ mice (5 controls and 6 with CCD). The donors for our colony of transgenic mice were originally provided by Dr. Xinzhong Dong's laboratory (Johns Hopkins University School of Medicine). Unoperated mice were housed 3 to 5 in a cage. After CCD surgery the operated mice were singly housed. The cages were individually ventilated, contained corn cob bedding and nesting material (for environmental enrichment). Food and an automated water supply were available ad lib. The housing facility maintained an air temperature of 22.2 ± 1.1°C, humidity of 50% (± 20%), and a 2:12 light dark cycle. The health and welfare of the animals were checked daily by veterinary technicians.

### Ethics statement

All our experimental procedures were carried out in the LaMotte laboratory, approved by the Institutional Animal Care and Use Committee of Yale University School of Medicine, and were in accordance with the guidelines provided by the National Institute of Health and the International Association for the Study of Pain.

### Behavioral testing

We measured pain-like behavior evoked by an indentation of the hairy skin of the dorsum of the hind paw with a von Frey filament that had a tip diameter of 200 μm and delivered a bending force of 20 mN. This stimulus was defined as nociceptive (painful) because it always elicited a brisk foot withdrawal when applied to the hind paw either to the plantar [13] or to the dorsal surface (present study). We presently observed that the shaking that occurred after foot withdrawal entailed either flapping the foot against the ground in a vertical motion, or at other times lifting the foot to the side and then shaking it without touching the ground. There was rarely any biting (scratching with the tooth) or licking behaviors directed toward the site of von Frey stimulation. We therefore used the incidence of shaking as a measure of hyperalgesia defined as increased pain-like behavior in response to a normally aversive stimulus (normally eliciting withdrawal).

The mouse was placed in an open-topped transparent plastic chamber (8x7x3.5 cm) mounted on a platform raised 7 cm from the lab bench. The animal was allowed to move freely around the chamber during training and testing. The walls of the test chamber were low enough to allow a maximal range of motion for the experimenter to apply the stimulus to the dorsum of the mouse's hind paw. The mouse occasionally looked over the side of the wall during exploration but was effectively deterred from escaping by the height of the chamber above the table below.

Stimuli were delivered from behind the mouse so the sight of the von Frey filament or the experimenter’s hand did not further disturb the mouse. Though stimuli were given in the same general area, repeated stimulation of the exact same location was avoided. The stimulus was not applied while the mouse was actively walking or grooming.

**Fig 1:**
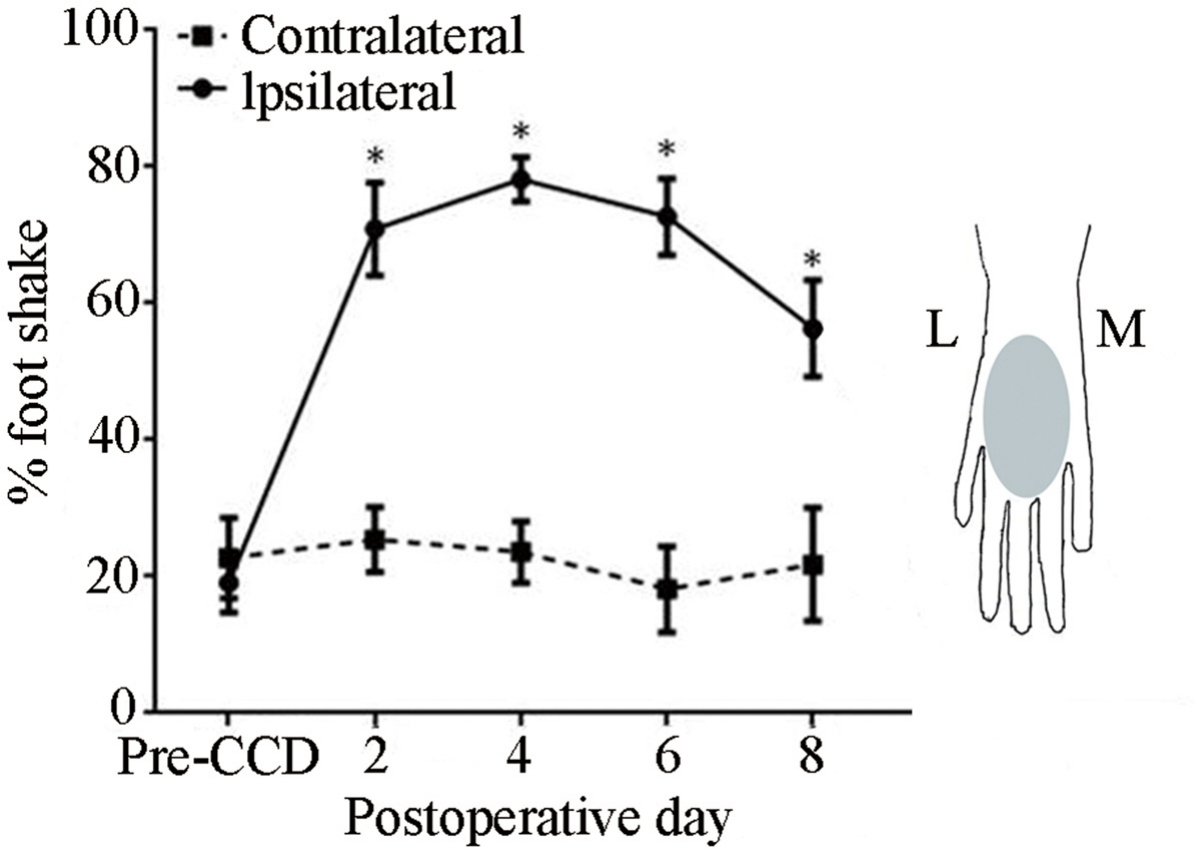
Effects of CCD on the incidence of foot-shaking evoked by punctate mechanical stimulation of the hairy skin. A von Frey filament with a tip diameter 200 μm, and delivering a bending force of 20 mN was applied to the dorsum of each foot before the CCD surgery ("Pre-CCD") and up to 8 days after the surgery. Mice (n = 11), *P < 0.05 indicates the statistical significance in comparisons of the mean incidence obtained for the foot ipsilateral to the CCD surgery (solid line) compared to both the contralateral foot (dashed line) and to the pre-operative ipsilateral baseline. Inset: Schematic diagram of the region where the von-Frey filament was applied (shaded area) on the hairy skin (dorsum) of the right hind paw. L: lateral, M: medial.

On each of 4 or 5 days before behavioral data were collected, each animal was habituated to the experimental setup for 30 to 60 min. The von Frey filament to be used for testing was periodically applied to the test site located on the middle of the dorsum of the hind paw (inset of Fig 1) within the L3 and L4 dermatomes [23, 24]. Behavioral responses were collected on two consecutive days before the surgery and on every other post-operative day beginning on day 1 or 2 and ending on day 7 or 8 after surgery. On each of these days, the mouse was allowed to acclimatize to the experimental setup for 30 min. During each test, each foot was indented with the 200-μm-diameter filament (evoking a withdrawal) while alternating between feet until each was stimulated 5 times. For each foot, the presence or absence of foot shaking in response to the stimulus was recorded and the result summarized as a percentage of the number of indentations.

### CCD Surgery

Under 1–2% isoflurane anesthesia, the intervertebral foramina of L3 and L4 were exposed and a L-shaped steel rod, 2 mm in length and 0.3 mm in diameter, was implanted into each foramen to compress each DRG [8, 10, 12].

A control group of mice received no surgical treatment. As for the possibility of sham surgeries, it was impractical or not possible to try to blind the experimenter(s) during electrophysiological recording as to the prior CCD surgery due to the appearance of the rods and their effects on the DRG. Previous studies found sham surgeries to have little effect on tactile allodynia or neuronal excitability in the rat [10, 11]. In a pilot study, we examined the effects of the surgical preparation without rod implantation on the incidence of spontaneous activity in cutaneous, mechanosensitive C-nociceptors. One week after this surgery, recordings were obtained, as presently described, from a total of 18 mechanosensitive C-nociceptive neurons each innervating the hairy skin of the hind paw. None of these neurons exhibited any signs of spontaneous activity. These neurons were not tested with all of the von Frey filaments used in the present experiments and thus not included in our data base. We did not explore any other effects of sham surgeries.

### In-vivo electrophysiological recording

In CCD mice, recording experiments were performed 5 to 7 days after the rod implantation. Under 1–2% isoflurane anesthesia, delivered via tracheostomy tube and maintained by a Kent Scientific "SomnoSuite" anesthesia system, a laminectomy was performed. The L3 DRG was fixed to a plate and superfused with warm artificial cerebrospinal fluid (ACSF) within a pool that was produced by sewing the skin to a ring [14, 20, 25]. The epineurium was removed and the cell bodies on the surface of the DRG observed at 40X under reflection microscopy. The cell bodies could also be viewed under epifluoresence for the purpose of identifying those that expressed MrgprA3 or, in other mice, MrgprD according to the presence of a GFP [25]. Both types of neurons have been identified as innervating the stratum granulosum of the epidermis [16, 19]. Collagenase P of 1 mg/ml (Roche Diagnostics, Indianapolis, IN, USA) was locally applied for 5 min to loosen neuronal cell bodies from their neighbors. A chosen cell body was drawn into the mouth of a glass micropipette (tip diameter, 20–25 μm) filled with ACSF and the occurrence of action potentials (APs) recorded extracellularly with a Multiclamp 700B amplifier (Molecular Device, Sunnyvale, CA). In the absence of external stimulation, if any APs occurred for more than 3 min, the neuron was classified as exhibiting spontaneous (ongoing) discharge provided the activity could not be attributed to normal activation of the nerve ending (e.g.—cool sensitive thermoreceptor) or any transient stimulation produced by application of the recording electrode.

We searched for and mapped the location of the neuron's cutaneous receptive field via application of various stimuli to the hind limb. These stimuli consisted first of gentle touch and pinching of the skin with the experimenter's fingers followed by more precise mapping with the application of a blunt glass probe and von Frey filament [9, 26]. A single heat stimulus consisting of a rapid temperature ramp from a base of 38°C to a 5 s plateau of 51°C and then back to the base was delivered by either a Peltier- or a chip-resistor thermode [14, 27]. Next, a series of 5 von Frey filaments were each applied to the receptive field for 1 s. The filaments had the same tip diameter 200 μm but differed in bending force. They were delivered in order of ascending forces of 2, 6, 20, 40, and 80 mN. The filament that delivered 20 mN was identical to the filament used to test the incidence of foot shaking in the behavioral testing.

At the end of each experiment, the neuron's axonal conduction velocity was measured by electrically stimulating the receptive field with two wire electrodes and then dividing the latency of the evoked AP by the distance between the receptive field and the cell body.

### Statistical analysis

Data are presented as mean ± SEM, and the criterion for significance for each statistical test was set at *P* < 0.05. For behavioral data, the incidence of foot shaking was statistically compared for the two hind paws using paired t tests for each day of testing and Bonferroni correction for multiple comparisons. For electrophysiological data, Fisher's Exact Test was used to determine the significance in differences in the incidence of spontaneous activity and in the proportion of neurons tested that were activated by a particular filament force for different neuronal types (MrgprA3^+^ and MrgprD^+^). The significance of differences in the mean number of APs evoked by each force in each neuronal type for control vs. CCD mice was tested with a two way ANOVA with repeated measures for force. This was followed by post hoc testing with Student’s t-test with a Holm-Bonferroni correction to the critical *P* value.

## Results

No spontaneous behaviors were observed in the mice before or during CCD such as foot lifting, shaking, nor were there abnormal behaviors directed toward the hind limb such as biting or licking. In tests of the incidence of responses to mechanical stimulation, the 20 mN filament would just noticeably bend and then nearly always elicit a foot withdrawal. This response was sometimes followed by a shaking of the foot beginning approximately a second after the onset of filament application.

For each foot, the percentage of applications that elicited a foot shaking was averaged for the responses obtained on the two days prior to performing the CCD surgery ("Pre CCD" in Fig 1). The mean percentage of filament applications that evoked foot shaking did not differ for the two feet prior to CCD. On the first postoperative test, the incidence of a foot shake significantly increased for the foot ipsilateral to the CCD injury but remained unchanged for the contralateral foot (paired t tests). The ipsilateral foot continued to exhibit a greater incidence of foot shaking than the contralateral foot up through day 8 (paired t tests and Fig 1).

**Fig 2:**
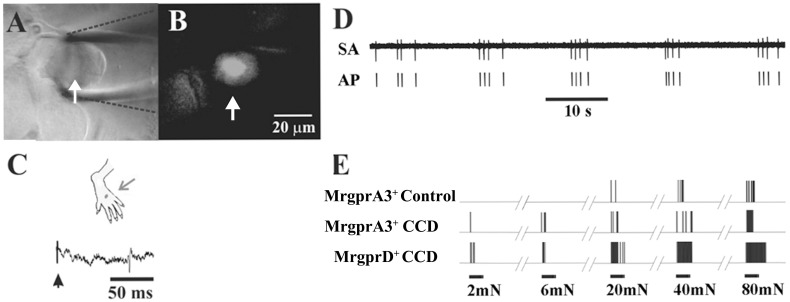
Spontaneous and mechanically evoked action potential activity electrophysiologically recorded in vivo from cell bodies of C-nociceptors. (A) Bright-field image of the cell body of a neuron whose action potentials are extracellularly recorded via a glass pipette electrode (outlined with dashed black lines) in the DRG of a control mouse. (B) Epifluorescent image of GFP indicated that the neuron expressed MrgprA3. The scale bar in B also applies to A. (C) The neuron was identified as a C-mechanoheat nociceptor (CHM) with a receptive field (RF) on the dorsum of the hairy skin (black dot) that was electrically stimulated (arrow) to obtain its conduction velocity (CV) (0.63 m/s). (D) Spontaneous activity of a CMH nociceptor recorded after CCD (RF on ankle, CV = 0.69 m/s). (E) Responses of three C-mechanosensitive nociceptive neurons to von Frey filaments each with the same tip diameter of 200-μm but delivering different bending forces of 2, 6, 20, 40, and 80 mN. Each also responded to noxious heat (responses not shown). These neurons include the neuron referred to in panels A-C ("MrgprA3+ control") and two neurons recorded from CCD mice, one expressing MrgprA3 with RF on the ankle and CV of 0.69 m/s ("MrgprA3 CCD") and the other expressing MrgprD+ with RF on 3rd toe and CV of 0.5 m/s ("MrgprD CCD").

Electrophysiological recordings were obtained from GFP-labeled neurons in four groups of mice: Nine unoperated "control mice" (4 that were MrgprA3^+^ and 5 MrgprD^+^) and from 11 "CCD mice" (5 MrgprA3^+^ and 6 MrgprD^+^) that had received the surgery 5 to 7 days earlier. The recording pipette was applied only to neurons selected on the basis of being readily accessible and also for exhibiting the GFP (Fig 2A and 2B). For control mice, AP recordings were obtained from 28 GFP-labeled neurons in unoperated control mice, 13 from the MrgprA3^+^ mice, and 15 obtained from the MrgprD^+^ mice. For CCD mice, AP recordings were obtained from 43 GFP labeled neurons, 15 from the MrgprA3^+^ mice and 28 from the MrgprD^+^ mice.

To test whether each neuron was spontaneously active we first recorded extracellularly from a GFP labeled cell body without delivering any stimuli to the animal for a period of at least 3 min. In control mice, both types of neurons were silent, i.e. exhibited no spontaneous activity, in agreement with previous findings [16, 19]. After CCD, 26.7% (4/15) of MrgprA3^+^ neurons and 32.1% (9/28) of MrgprD^+^ neurons exhibited SA (Fig 2C). The proportions of spontaneous vs. silent neurons were not significantly different for the two types of neurons but each was significantly greater after CCD vs. under control conditions (Fisher's Exact tests). The median SA discharge rates (number of APs/180 s) were similar for the MrgprA3^+^ and MrgprD^+^ neurons (1.16 and 1.13 Hz, respectively). The receptive fields were subsequently identified for three of the four MrgprA3^+^ neurons exhibiting SA but for only one of the of the nine MrgprD^+^ neurons.

**Fig 3:**
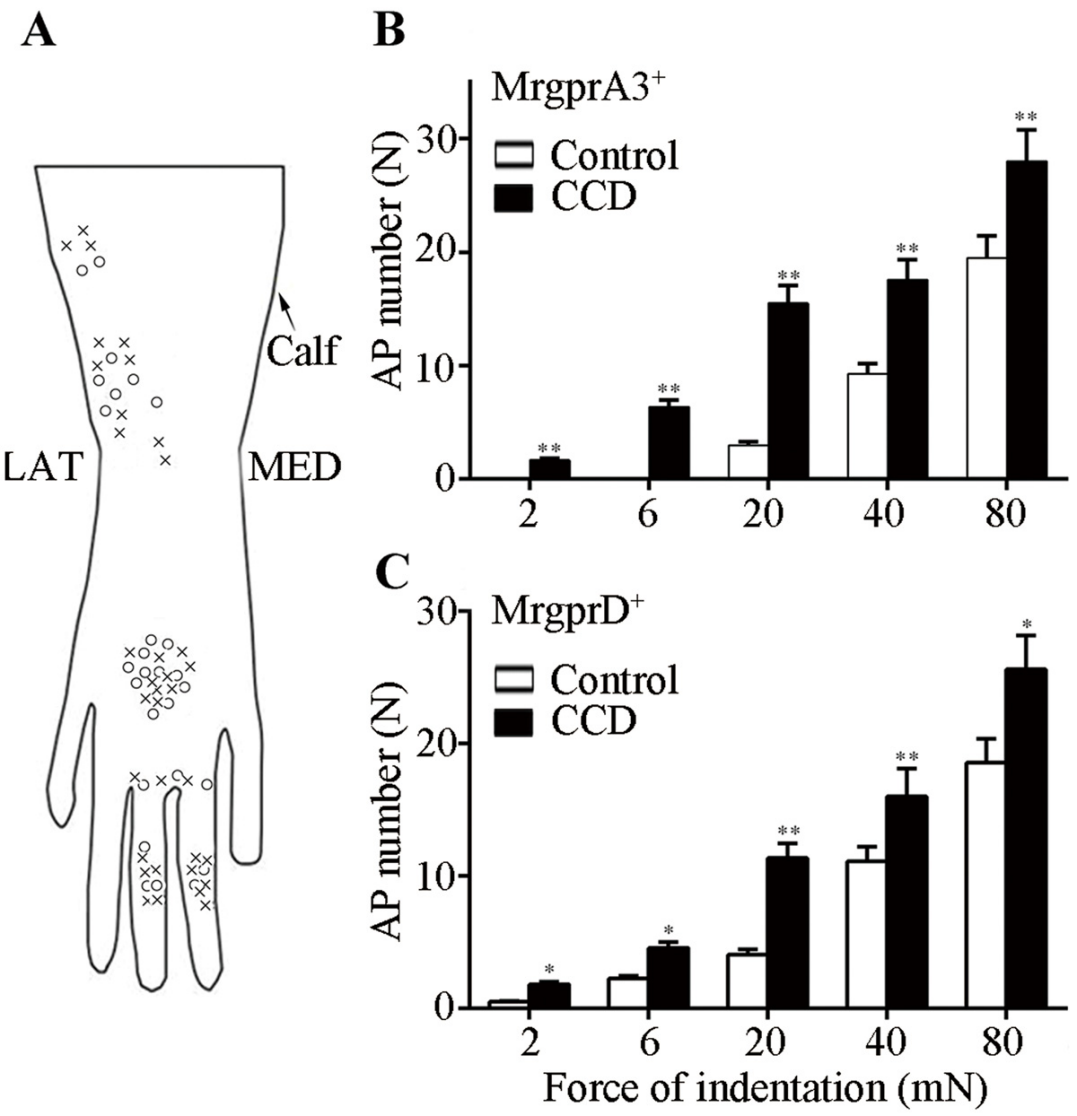
Responses of nociceptors to punctate mechanical stimulation as a function of force of indentation. Von Frey filaments (200 μm tip diameter) with differing bending forces (2-80mN) were each applied to the receptive field for 1 s. (A) Schematic diagram of the locations of receptive fields for each of the MrgprA3+ (circles) and MrgprD+ (crosses) expressing nociceptors on the dorsum (hairy) skin of the hind paw. L: lateral, M: medial. (B) Mean number of action potentials evoked in MrgprA3+ neurons, control mice (n = 13, open bars) and CCD mice (n = 14, closed bars). (C) Mean number of action potentials evoked in MrgprD+ neurons from control- (n = 15) and CCD mice (n = 20) (*P<0.05, **P<0.01).

The neurons with SA and receptive fields and the other GFP labeled neurons that did not have SA and included in the present study were all identified as mechanosensitive nociceptors with C-fibers innervating the hairy skin of the lower hind leg or dorsum of the foot (Figs 2D and 3A). The mean axonal conduction velocity for all neurons tested was 0.62 ± 0.01 m/s (n = 62) and was not significantly different for CCD vs. control or MrgprA3^+^ vs. MrgprD^+^ neurons.

Each neuron responded with increasing discharges to increasing forces of indentation with the 200-μm von-Frey filaments and some neurons. Some neurons were also responsive to the noxious heat stimulus of 51°C. The heat responsive neurons (CMH polymodal nociceptors), included 9 MrgprA3^+^ neurons (4 control, 5 CCD) and 9 MrgprD^+^ neurons (5 control and 4 CCD).

The number of APs evoked by each force of indentation was averaged separately for neurons obtained from MrgprA3^+^ and MrgprD^+^ mice and compared for the two experimental treatments (control and CCD). For control mice, mechanically evoked APs were recorded from 28 GFP-labeled neurons in unoperated control mice, 13 from the MrgprA3^+^ mice, and 15 obtained from the MrgprD^+^ mice. For CCD mice, mechanically evoked APs were obtained from 34 GFP labeled neurons, 14 from the MrgprA3^+^ mice and 20 from the MrgprD^+^ mice. For both types of Mrgpr^+^ neurons, the mean number of APs per stimulus increased significantly with force, and was significantly greater for CCD- than for control treatments (RMANOVA with 2 treatments × 2 transgenic types × 5 forces, Fig 3B and 3C). There were no significant differences in the responses of the two types of Mrgpr^+^ neurons. Consequently, to increase the sample size of these C-nociceptors in subsequent analyses, the data for both types were combined.

For the combined responses of both neuronal types, and in response to each force (2 mN to 80 mN) the mean number of APs was significantly greater after CCD than under control conditions (post hoc testing with Student’s t-test and a Holm-Bonferroni correction).

An additional measure of the magnitude of response to each force of indentation besides the mean number of APs is the proportion of nociceptors that were activated. We investigated whether the 20 mN-filament that elicited a greater incidence of aversive behaviors after CCD also activated a greater proportion of mechanosensitive C-nociceptors tested. Of the total number of Mrgpr^+^ neurons tested, the proportion responsive to the 20 mN filament was significantly greater after CCD than for unoperated controls (21/21 vs. 16/25 respectively, Fisher's Exact Test). Thus, the stimulus that elicited hyperalgesia after CCD also elicited a greater number of APs and a greater number of mechanosensitive C-nociceptors. In addition, a significantly greater proportion of Mrgpr^+^ neurons were activated by forces of 2 and 6 mN after CCD (vs. control) but not by the higher forces of 40 and 80 mN.

**Fig 4:**
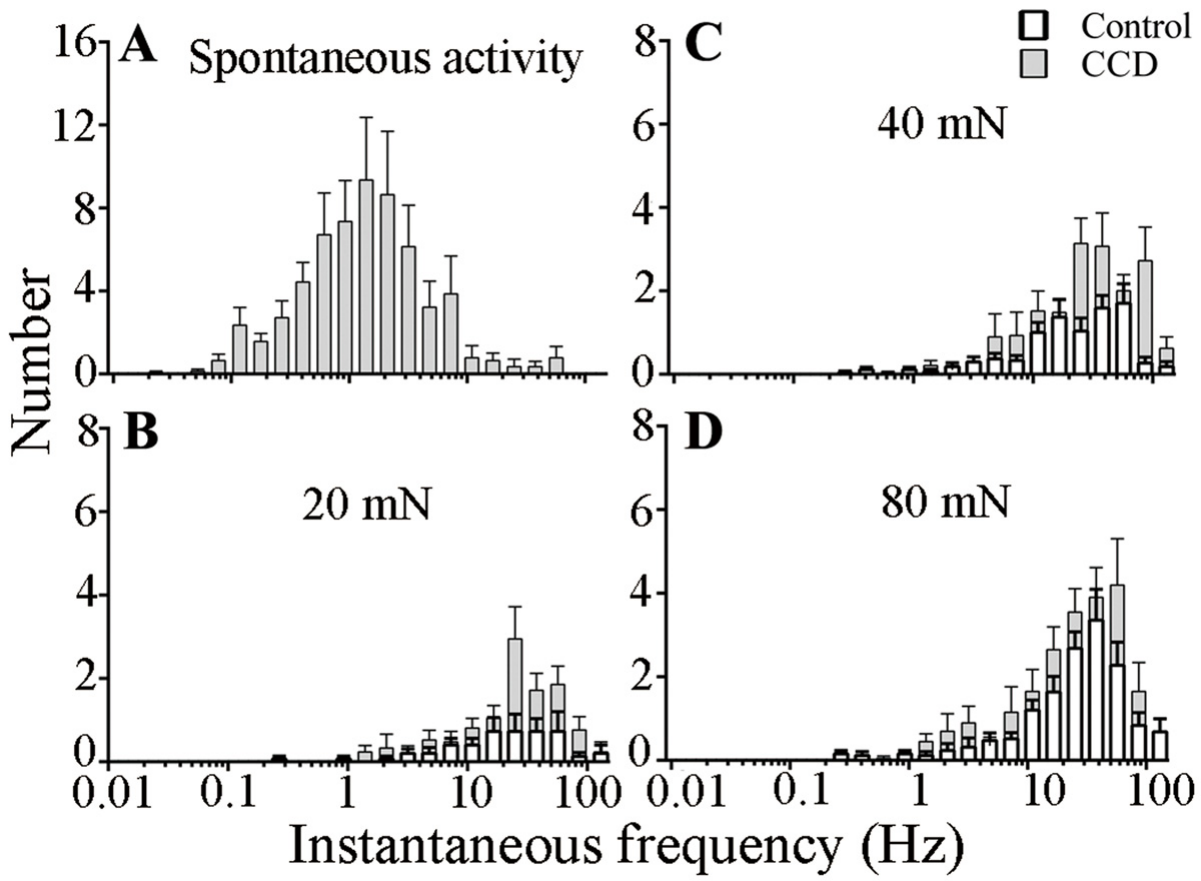
Histograms of instantaneous frequencies of spontaneous and mechanically evoked responses of C mechanosensitive nociceptors. The number of instantaneous frequencies that occurred within each bin (log increments, factor √2) is combined for MrgprA3+ and MrgprD+ nociceptors and plotted for control mice (closed bars, n = 28) and CCD mice (open bars, n = 34). (A) Spontaneous activity, observed only after CCD. (B–D) Responses to 20, 40 and 80 mN forces delivered by von Frey filaments each with the same tip diameter of 200 μm.

The temporal pattern of discharge in the population of mechanosensitive C-nociceptors also conveys information related to the intensity of stimulation, pain-like behavior, and hyperalgesia. Presumably, if similar patterns of discharge occurred, even on occasion, in the absence of mechanical stimulation, i.e. within the SA, they might be associated with an aversive behavior similar to that evoked by the punctate stimulation. Instantaneous frequency histograms were generated from the discharges of all Mrgpr neurons with SA after CCD (Fig 4A) and the discharges evoked in all the Mrgpr neurons activated by the filament of 20 mN (Fig 4B) and higher forces of 40 and 80 mN (Fig 4C and 4D) in control vs. CCD mice. For both CCD and control neurons, the distributions of discharge frequencies by the aversive mechanical stimuli were shifted well to the right of those occurring spontaneously after CCD. The median instantaneous frequency of SA (1.1 Hz) was about 20 times less than the median frequencies evoked by the 20 mN (22.9 Hz) and higher forces of indentation (29.7 and 23.4 Hz for 40 and 80 mN, respectively).

Although mechanically evoked discharges in CCD neurons sometimes outlasted the duration of the von Frey stimulation, there were no statistically significant differences in the mean durations of AP discharges in response to von-Frey filament forces of 20, 40, and 80 mN between CCD- and control neurons of either transgenic type (RMANOVA with 2 treatments × 2 transgenic types × 3 forces).

## Discussion

Previous findings established that CCD resulted in behavioral signs of allodynia to punctate von Frey stimuli [10]. As in models of nerve injury or inflammation, the allodynia was manifested as an increased incidence of foot withdrawal to punctate stimuli that did not elicit this aversive response prior to the injury. We now show that CCD also produces mechanical hyperalgesia, measured as an increased incidence of foot shaking to a normally aversive punctate stimulus.

A methodological problem described for rodent models of nerve injury, has been that aversive behavioral responses to punctate mechanical stimuli often cannot be measured prior to injury when higher bending forces are achieved by the use of stiffer filaments with wider tip diameters. For example, stiffer filaments delivered to plantar skin of the rat could lift the foot under control conditions without eliciting a withdrawal [28, 29]. One approach is to prick the skin with a needle, a stimulus that always elicits a withdrawal, and then using an increase in the duration of foot lifting as a measure of the mechanical hyperalgesia resulting from a nerve injury [30, 31].

The aversive mechanical stimuli applied in the present approach were von Frey type filaments of the same tip diameter of 200 μm that, depending on bending force, were previously found to elicit different intensities of pain when applied to hairy skin of the hand in human [32] and foot withdrawal when applied to the plantar surface of the foot of healthy, control mice [13]. Applied in the present experiments to the receptive fields of mechanosensitive C-nociceptors in the hairy skin, these stimuli elicited discharges that were graded with force. After CCD, the same stimuli elicited a greater number of action potentials in response to each force and, at the lower forces recruited a greater proportion of activated nociceptors. The filament and force that elicited a greater incidence of foot shaking after CCD also activated a greater proportion of C nociceptors and elicited a greater number of action potentials. Thus, a compressive injury applied to the DRG can cause an increase in the excitability of the cutaneous terminals of C fibers with mechanosensitive nociceptors.

There are limitations to the present study. First, the behavioral tester and the electrophysiologists were not blinded because of the obvious laterality of the pain behavior and the appearance of the operated DRG, respectively.

Second, aside from a pilot study that yielded no effects, extensive sham operations were not undertaken because in a previous study conducted in rats, a transient insertion of rods without implantation resulted in neither sustained tactile allodynia nor neuronal hyperexcitability [10, 11]. Further investigation would be required to determine whether this type of sham surgery or other types that do not include rod implantation might contribute to any of the CCD induced effects presently observed.

Another limitation is that in both the electrophysiological and the behavioral types of experiment the rate and duration of force delivery were subject to small variations upon repetition because von-Frey type stimuli were applied by hand. But because such stimuli are commonly used for behavioral testing we thought it advantageous for comparisons of nociceptor responses and behavior to use the same type of stimulus in both experiments. In the future, it may be possible to design a robotic device that could indent the dorsum of the foot with servo control of force in both the awake-behaving and anesthetized mouse.

In axotomy models of neuropathic pain, there is evidence for increased discharges of intact, nociceptive neurons to von Frey stimuli that elicit tactile allodynia after the transection of spinal nerves in rats [33] or distal peripheral nerves in mice [34]. The mechanosensitive neurons that remain intact after nerve injuries include those with C fibers but also some with thinly-myelinated A-fibers (A-nociceptors). A future direction would be to test whether the cutaneous A-nociceptors that are activated by the aversive punctate stimuli presently used would exhibit enhanced discharges after CCD.

In addition to the peripheral sensitization of nociceptors, as a cause of punctate hyperalgesia, CCD might also cause a central sensitization of nociceptive neurons in the spinal dorsal horn. For example, SA originating from the compressed ganglion [13, 20] and occurring in certain C-nociceptors (not necessarily only the ones we recorded here) might enhance the excitability of central neurons that receive a convergent input from both low-threshold and nociceptive cutaneous mechanoreceptors [35].

But could the SA we observed in mechanosensitive C-nociceptors expressing MrgprA3 or MrgprD cause a central neuronal sensitization that leads to punctate mechanical hyperalgesia? The distribution of instantaneous frequencies of discharge in the SA discharge in Fig 4A is quite similar to that recorded from a) C-mechanoheat sensitive cutaneous nociceptors in response to the pruritic agent cowhage in the mouse [20] and in the monkey [21], b) MrgprA3^+^ and MrgprD^+^ neurons in response to injection of chemical pruritogen in mice [16, 19] and unpublished response analyses, and c) the spontaneous discharge of MrgprA3^+^ neurons innervating an area of allergic contact dermatitis (median IF of 1.0 Hz) [25]. These pruritic agents elicit itch and minor nociceptive sensations in human and site-directed itch- and pain-like behaviors in the mouse. Also, in human there is sometimes an area of secondary punctate hyperalgesia and hyperknesis in the skin surrounding the site of pruritic chemical application [36, 37] providing evidence for central sensitization. However, these areas of enhanced mechanical sensitivity are not observed in the absence of chemically evoked sensation [36] and, in the present study we found no evidence for spontaneous site-directed behaviors after CCD such foot lifting, biting or licking.

In rats with combined axotomy and inflammation of spinal nerves, spontaneous foot lifting was correlated with the spontaneous firing of C-nociceptors, some identified as cutaneous, which exhibited a median discharge rate of 1.8 Hz [38, 39]. The CCD-induced SA we observed in mice had a lesser median discharge rate of 1 Hz. This rate would not seem likely to elicit spontaneous foot lifting and shaking or other aversive behaviors because the instantaneous frequencies are much lower than those associated with the aversive responses elicited by the aversive punctate stimulus of 20 mN or higher forces (Fig 4).

The higher instantaneous frequencies of discharge evoked by algesic stimuli in comparison to pruritic provides a mechanism for the differential neural coding of pain and itch within the discharges of the same population of mechanosensitive cutaneous nociceptors. Such a mechanism may also apply to the differential responses of mechanically insensitive C-nociceptors which exhibit higher instantaneous discharge frequencies to cutaneous injections of capsaicin which is painful than to histamine which is itchy [40].

But because the CCD-induced SA discharges in the mechanosensitive C-nociceptors are not unlike those elicited in these same nociceptors by pruritic chemicals one might ask why there were not some spontaneous itch-like (and pain-like) behaviors directed toward the ipsilateral hind limb. Perhaps one reason is that the central pruriceptive or nociceptive effects of SA in cutaneous C-nociceptors were blocked by central inhibitory neurons activated by simultaneous SA known to be generated from the compressed DRG in large diameter mechanosensitive neurons [9]. Presumably, the site-directed behaviors elicited by a pruritic chemical would be associated with activity in only a small proportion of cutaneous C-nociceptors and then in the absence of concomitant activity in large diameter mechanosensitive neurons. Electrophysiological recordings from projection neurons in the spinal dorsal horn in the future might reveal the cellular mechanisms of nociceptive or pruriceptive transmission and afferent inhibition after CCD.

Although CCD did not result in the production of spontaneous site-directed behaviors involving the hind paw in the mouse, certain types of neuropathic itch, in humans, might be caused by a neuronal compression injury of cervical ganglia or nerves (brachioradial pruritus) or the dorsal rami of thoracic spinal nerves (notalgia paresthetica) [37, 41]). In these cases perhaps there are conditions of compression and inflammation that selectively activate pruriceptive cutaneous nociceptors without producing similar effects on the non-pruriceptive nociceptive or non-nociceptive sensory neurons. These possibilities remain to be explored in animal models.

## Supporting Information

S1 Checklist (DOCX)
