# Supplementary material for: Chronic Compression of the Dorsal Root Ganglion Enhances Mechanically Evoked Pain Behavior and the Activity of Cutaneous Nociceptors in Mice
Source: PLoS One. 2015 Sep 10;10(9):e0137512. doi: 10.1371/journal.pone.0137512 (PMC4565551; doi:10.1371/journal.pone.0137512)

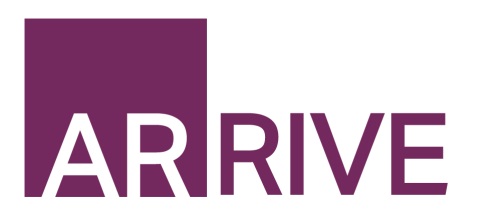


The ARRIVE Guidelines Checklist

Animal Research: Reporting In Vivo Experiments

Carol Kilkenny^1^, William J Browne^2^, Innes C Cuthill^3^, Michael Emerson^4^ and Douglas G Altman^5^

*^1^The National Centre for the Replacement, Refinement and Reduction of Animals in Research, London, UK, ^2^School of Veterinary Science, University of Bristol, Bristol, UK, ^3^School of Biological Sciences, University of Bristol, Bristol, UK, ^4^National Heart and Lung Institute, Imperial College London, UK, ^5^Centre for Statistics in Medicine, University of Oxford, Oxford, UK.*

|  | | ITEM | RECOMMENDATION | Section/ Paragraph |
| --- | --- | --- | --- | --- |
| 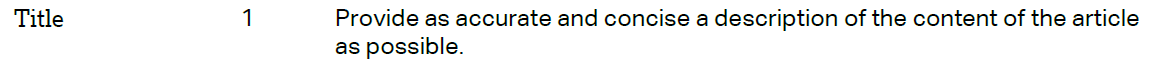 | | | title |  |
| 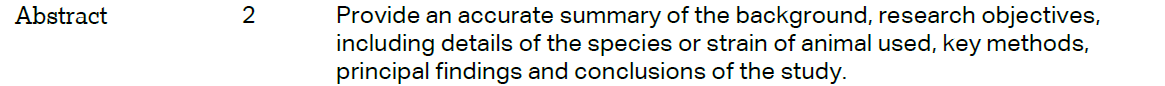 | | | abstract |  |
| INTRODUCTION | | |  |  |
| 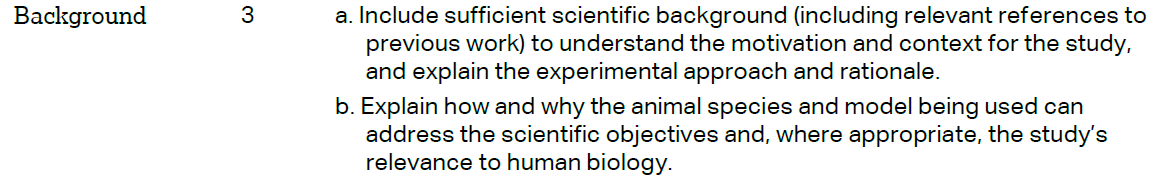 | | | Para-graphs1-3  Para-  graphs 2 and 4 |  |
| 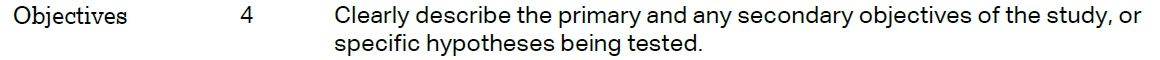 | | | Para. 1,4,5 |  |
| METHODS | | |  |  |
| 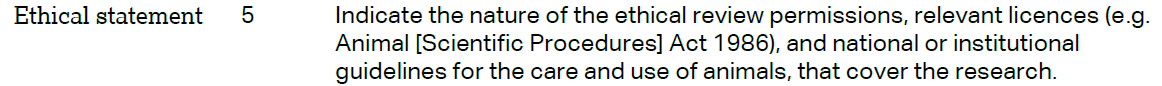 | | | Para. 2 |  |
| 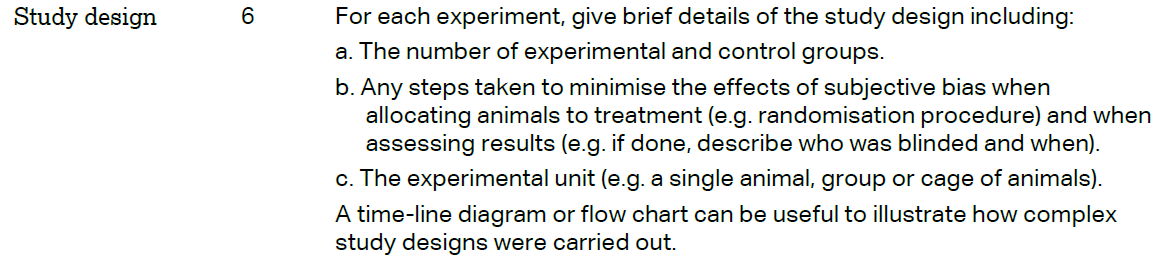 | | | Para 1,5 |  |
| 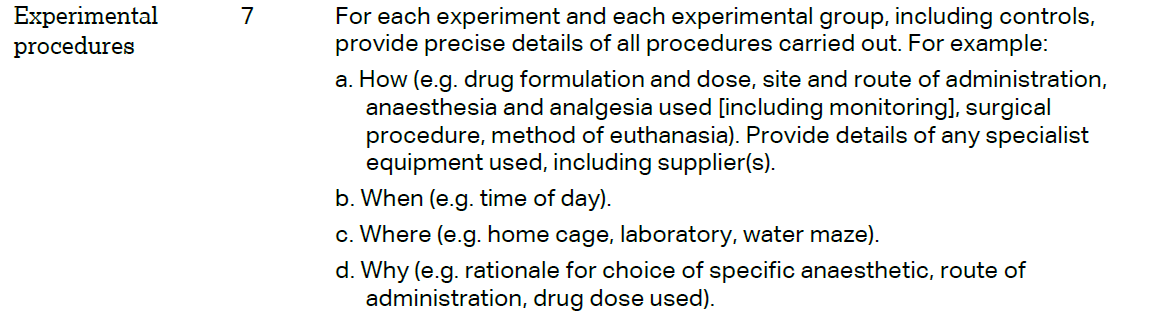 | | | Para 6-7 |  |
| 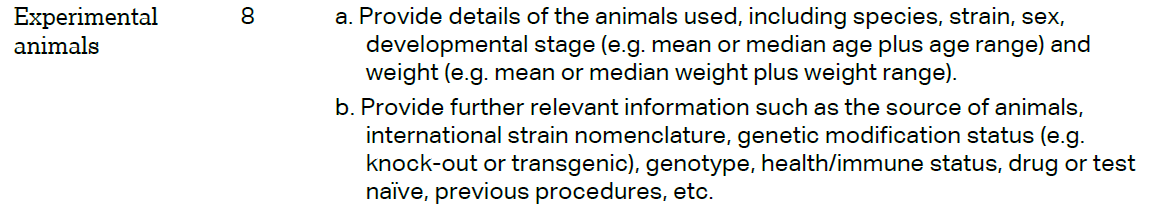 | | | Para 1 |  |

The ARRIVE guidelines. Originally published in *PLoS Biology*, June 2010^1^

| 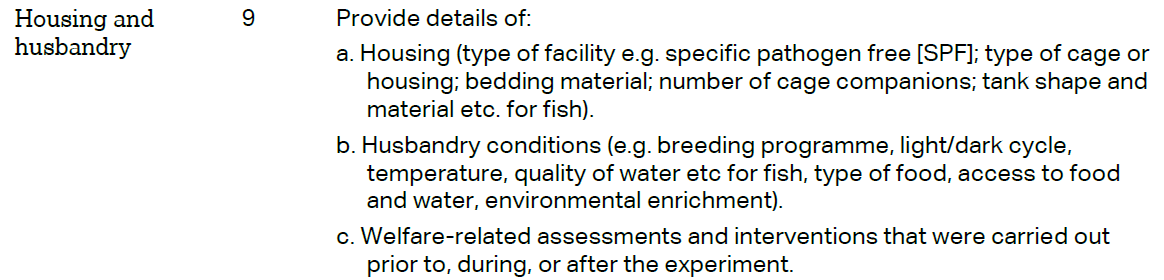 | Para 1 | |
| --- | --- | --- |
| 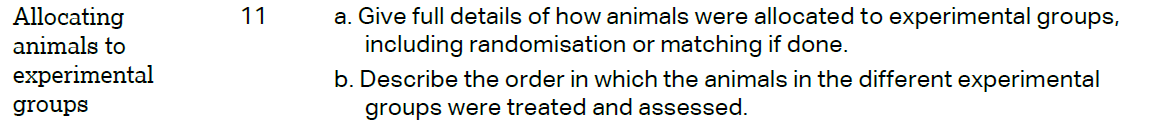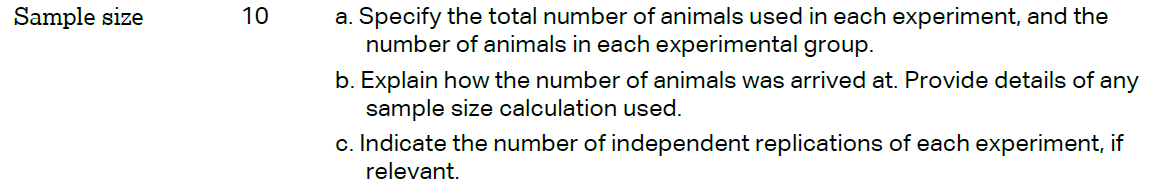 | Para 1 | |
|  |  | |
| 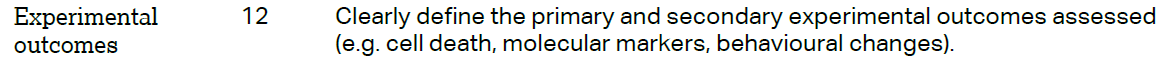 |  | |
| 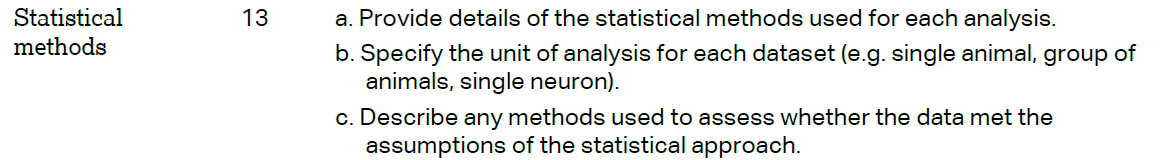 | Para 9 | |
| RESULTS |  | |
| 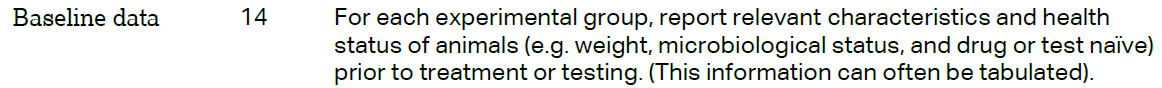 | Methods Para 1 | |
| 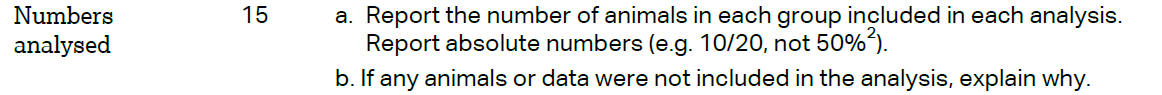 | Para 3,4 | |
| 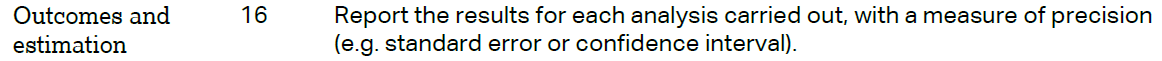 | Para 2 & Fig. 1, para 6, para 9, Fig.3, para 11-12, Fig. 4 | |
| 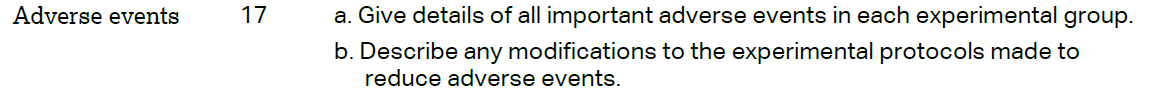 | There were no adverse events | |
| DISCUSSION |  | |
| 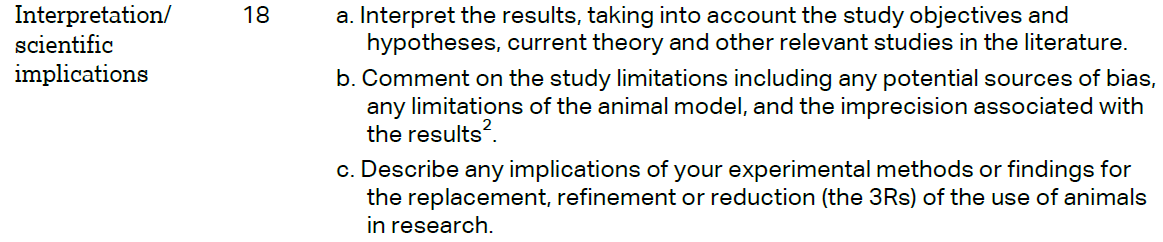 | Para 1- 4  Para 4  There are none | |
| 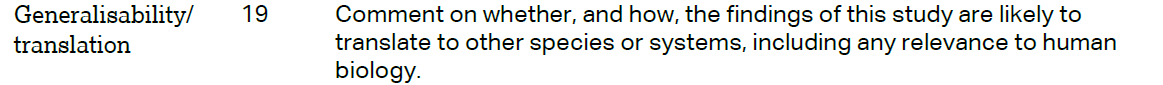 | Para 10 | |
| 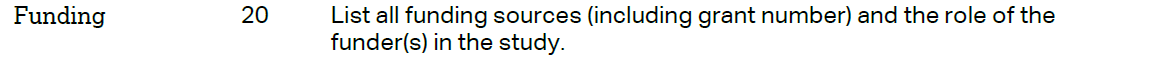 | | Listed in designated space |


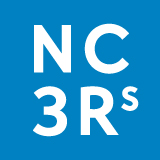

Supplement: S1 Checklist — (DOCX) [file pone.0137512.s001.docx]
